# The Treatment of Diseases by the Hypodermic Method

**Published:** 1879-12

**Authors:** 


The Treatment of Diseases by the Hypodermic Method—A Manual of Hypodermic Medication. By Robert Bartholow, M.A., M.D., L.L.D., Professor of Materia Medica and General Therapeutics in the Jefferson Medical College of Philadelphia. Third Edition Enlarged. J. B. Lippincott & Co, Philadelphia. 1897.

This is a neat volume of two hundred and fifty pages and devoted to the important subject of hypodermic medication. All the facts and observations connected with this practice are given in detail, and will repay any one the time spent in its perusal.

